# Complete PacBio Single-Molecule Real-Time Sequence of a Novel Probiotic-Like Bacterium, Rouxiella badensis subsp. *acadiensis*, Isolated from the Biota of Wild Blueberries in the Acadian Forest

**DOI:** 10.1128/mra.01340-22

**Published:** 2023-04-13

**Authors:** Elisa Salvetti, Julien Tremblay, Melanie Arbour, Jean-François Mallet, Luke Masson, Chantal Matar

**Affiliations:** a Department of Biotechnology, University of Verona, Verona, Italy; b Energy, Mining and Environment Research Center, National Research Council Canada, Montreal, Quebec, Canada; c Human Health Therapeutics Research Center, National Research Council Canada, Montreal, Quebec, Canada; d Department of Cellular and Molecular Medicine, Faculty of Medicine, University of Ottawa, Ottawa, Ontario, Canada; e School of Nutrition Sciences, Faculty of Health Sciences, University of Ottawa, Ottawa, Ontario, Canada; University of Arizona

## Abstract

The PacBio whole-genome sequencing of the potential probiotic strain Canan SV-53T recovered from lowbush blueberries demonstrates high homology to Rouxiella badensis but with distinct enough clusters to propose the name Rouxiella badensis subsp. *acadiensis*. The sequencing also detected the presence of two native plasmids.

## ANNOUNCEMENT

Strain Canan SV-53^T^ was isolated in 2003 from the surface of lowbush blueberries (Vaccinium angustifolium Aiton) in the Acadian Forest of New Brunswick, Canada. The isolation was the result of a project aimed at selecting bacteria from the biota of wild berries with the ability to significantly increase the antioxidant potential of fermented berries (1, 2).

To isolate the strains, blueberries were washed with tryptic soy broth (TSB), and the washing solutions were then plated on tryptic soy agar (TSA) at 25°C for 36 h (1). Colonies were grown in TSB, and each culture was used to inoculate filter-sterilized blueberry juice. SV-53^T^ showed the highest antioxidant capacity after fermentation and as such was further characterized (1).

At the time of isolation, analysis of the partial 16S rRNA gene sequence placed SV-53^T^ within the family *Enterobacteriaceae*, but a formal description was not made. Later, the sequence was reanalyzed, and a match was found for a newly described species, Rouxiella badensis (3, 4). Whole-genome shotgun sequencing and assembly of SV-53^T^ were performed to determine the phylogenetic position of the isolate. The annotated genome was also screened for known virulent or toxic compounds, so that the strain could be classified as safe candidate for human consumption as a probiotic. The bacterium has been deposited at the International Depository Authority of Canada (160103) and at the American Type Culture Collection Patent Depository (PTA-126681).

A single colony of SV-53^T^ was cultured in potato dextrose broth at 22°C for 18 to 20 h. Genomic DNA was extracted from 2 mL of pure culture using the Gentra Puregene yeast/bacteria kit (Qiagen) and was sent to the Genome Quebec and McGill University Innovation Centre. The DNA was used to prepare a sheared large-insert library, which was processed on the Pacific Biosciences (PacBio) RS II single-molecule real-time (SMRT) platform.

With a mean length of 7,016 bases, the 157,947 subreads totaled 1,108,175,630 bases. The sequencing facility used the Hierarchical Genome Assembly Process (HGAP) workflow to assemble the contigs. The sequencing produced three circular contigs, which included a chromosome (4,932,173 bp) and two native plasmids (pUnnamed1, 120,691 bp; pUnnamed2, 82,584 bp). The contigs have GC contents of 53% for the chromosome, 49.4% for pUnnamed1, and 44.2% for pUnnamed2. Each contig was annotated via the NCBI Prokaryotic Genome Annotation Pipeline version 4.12 using GeneMarkS-2-1.10 (5).

To confirm the affiliation of SV-53^T^ to the species *R. badensis*, the average nucleotide identity (ANI) was computed (http://enve-omics.ce.gatech.edu/ani/index) using default BLAST parameters. The two-way ANI value between SV-53^T^ and *R. badensis* SR3 was 99.69%, well above the species threshold of 95% ANI (6).

In a comparison of KEGG ortholog functions with *R. badensis* SR3, *R. badensis* subsp. *acadiensis* was found to be enriched in several functions, such as genes involved in secretion systems and prokaryotic defense systems. No known virulence genes were found in SV-53^T^.

### Data availability.

The complete genome of *R. badensis* subsp. *acadiensis* C173 (Canan SV-53^T^) has been deposited at NCBI under the GenBank accession no. CP060592. The pUnnamed 1 and pUnnamed2 plasmid sequences have been deposited at GenBank under the accession no. CP060593 and CP060594, respectively. The raw sequencing data can be found under SRA accession no. PRJNA648847.
